# Methyltransferase-like 3 is a target for the diagnose and therapy of clear cell renal carcinoma

**DOI:** 10.3389/fphar.2025.1534655

**Published:** 2025-04-17

**Authors:** Dongqiong Xiao, Xiaojuan Su

**Affiliations:** Department of Emergency, Key Laboratory of Birth Defects and Related Diseases of Women and Children (Ministry of Education), West China Second University Hospital, Sichuan University, Chengdu, China

**Keywords:** methyltransferase-like 3, clear cell renal carcinoma, pathogenesis, drug resistance, diagnosis biomarker, therapeutic target

## Abstract

Patients diagnosed with clear cell renal carcinoma (ccRCC) frequently exhibit metastatic disease, which complicates treatment strategies, underscoring the urgent need for mechanistic insights and early diagnostic biomarkers. Current research is dedicated to uncovering the mechanisms behind ccRCC development and resistance to treatment, with a particular focus on the role of methyltransferase-like 3 (METTL3) in RNA N^6^-methyladenosine modification, a key gene regulatory process. This review synthesizes current evidence on METTL3’s functions, revealing its oncogenic activity through m^6^A-mediated regulation of RNA stability and translation, which promotes tumor progression, metastasis, and chemoresistance. We further explore METTL3’s dual diagnostic and therapeutic relevance, including its utility as a prognostic biomarker and its targeting via novel strategies such as small-molecule inhibitors (e.g., *Erianin*) and combination therapies with mTOR or immune checkpoint inhibitors. By consolidating these advances, this review positions METTL3 as a critical node for advancing precision medicine in ccRCC.

## 1 Introduction

Clear cell renal carcinoma (ccRCC) constitutes about 85% of all diagnosed cases of RCC, yet early detection remains challenging due to the absence of reliable biomarkers (Siegel et al., 2023). Metastatic disease, a key driver of mortality in ccRCC, correlates with a 5-year survival rate below 10%, reflecting the tumor’s molecular and clinical heterogeneity (Siegel et al., 2023). Unraveling the mechanisms underlying ccRCC progression and therapy resistance is critical, particularly through emerging insights into RNA N^6^-methyladenosine (m^6^A) modification—a dynamic regulator of post-transcriptional gene expression (Sendinc and Shi, 2023).

The m^6^A modification fine-tunes RNA stability, localization, and translation through the coordinated efforts of various components, including writers, erasers, and readers, subsequently affecting gene expression across various biological processes (Boulias and Greer, 2023). The addition of the m^6^A marks is carried out by writer proteins, primarily the methyltransferase-like 3/14 (METTL3/14) complex, along with several other associated proteins (Xu et al., 2023). Within this complex, METTL3 acts as the enzymatically active unit, while METTL14 provides structural support essential for RNA binding and the stability of the entire assembly (Xu et al., 2023). Conversely, the role of erasers is to eliminate the methylation modifications present in RNAs (Meyer and Jaffrey, 2017). Additionally, the ultimate fate of target RNAs is significantly influenced by the recognition of m^6^A reader proteins, including those from the YT521-B homology domain-containing family (YTHDF), YTH domain-containing family (YTHDC), and insulin-like growth factor 2 mRNA binding protein family (IGF2BP) (Flamand et al., 2023).

METTL3, the catalytic subunit responsible for modifying RNA m^6^A, plays a crucial role in methylating adenine residues in post-transcriptional RNAs (Wang et al., 2016; Du et al., 2018). Dysregulation of METTL3 has emerged as a hallmark of ccRCC, where it drives oncogenesis through methyltransferase-dependent RNA modification (e.g., enhancing pro-tumorigenic mRNA translation) and methyltransferase-independent signaling (e.g., activating PI3K/AKT pathways) (Mao et al., 2022; Luo et al., 2020; Dou et al., 2023; Schöller et al., 2018; Zeng et al., 2024). These mechanisms promote tumor proliferation, metastasis, and chemoresistance, positioning METTL3 as a pivotal therapeutic target.

Despite growing interest in METTL3’s role in ccRCC, a comprehensive synthesis of its context-specific functions, clinical relevance, and therapeutic targeting is lacking. This review bridges this gap by systematically analyzing METTL3’s mechanistic contributions to ccRCC pathogenesis and its potential as a diagnostic biomarker and therapeutic node. We further evaluate emerging strategies to inhibit METTL3, including small-molecule agents and combination therapies, offering a roadmap for advancing precision medicine in ccRCC.

## 2 Insights for the roles and mechanisms of METTL3 in ccRCC

Numerous studies have underscored the oncogenic function of METTL3 in human ccRCC, emphasizing its crucial role in tumor progression and prognostic evaluation. For instance, Chen et al. (Chen et al., 2020) found that a signature comprising METTL3 and METTL14 can independently predict risks, effectively distinguishing between patients with ccRCC. Furthermore, elevated levels of METTL3 have been consistently detected in ccRCC tissues (Gundert et al., 2021). Analyses of risk signatures among patients with ccRCC reveal a strong correlation between increased METTL3 expression and adverse pathological characteristics (such as stage, grade, and clinical classifications T, N, and M), along with survival outcomes, which highlights its potential utility in ccRCC prognosis (Gundert et al., 2021).

### 2.1 METTL3 and ccRCC pathogenesis

#### 2.1.1 METTL3 functions by regulating tumor-related mRNAs

**FIGURE 1:**
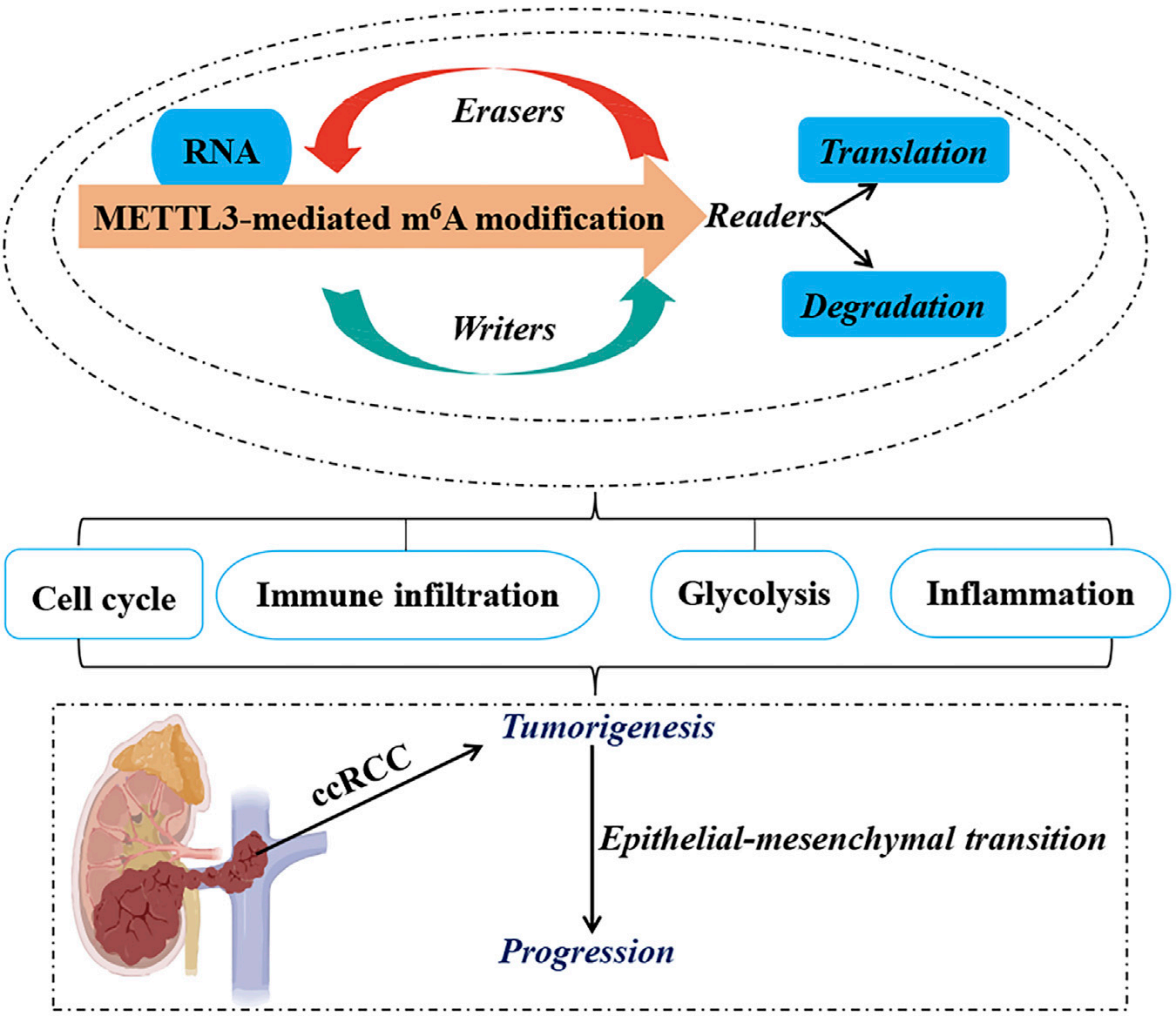
Functional Roles of METTL3 in ccRCC. The RNA m^6^A modification mediated by METTL3 plays a pivotal role in the oncogenesis, epithelial-mesenchymal transition, and advancement of ccRCC by coordinating various cellular mechanisms, such as the modulation of cell cycle progression, lipid metabolic pathways, immune cell infiltration, and evasion, as well as the regulation of glycolytic metabolism. Methyltransferase-like 3 (METTL3). N^6^-methyladenosine (m^6^A). Clear cell renal carcinoma (ccRCC).

**FIGURE 2:**
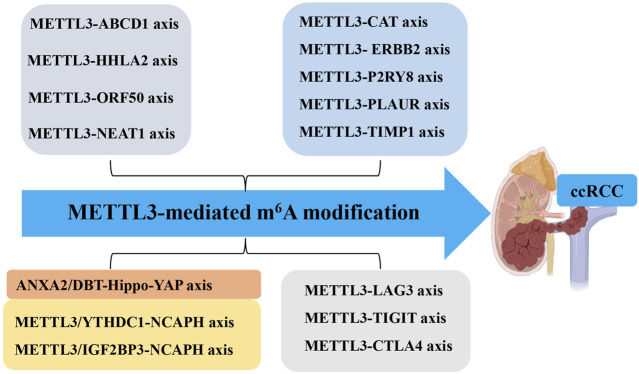
Molecular Mechanisms of METTL3 in ccRCC. The molecular mechanisms of METTL3 in ccRCC include different METTL3-RNA axes. Methyltransferase-like 3 (METTL3). N^6^-methyladenosine (m^6^A). Clear cell renal carcinoma (ccRCC). ATP-binding cassette subfamily D member 1 (ABCD1). Open reading frame 50 (ORF50). On-SMC condensin I complex subunit H (NCAPH). YTH N^6^-methyladenosine RNA binding protein (YTHDF). Dihydrolipoamide branched chain transacylase E2 (DBT). Yes1-associated transcriptional regulator (YAP). Nuclear enriched abundant transcript 1 (NEAT1).

METTL3 plays a significant role in regulating tumor mRNAs within ccRCC. For instance, the METTL3-ATP-binding cassette subfamily D member 1 (ABCD1) axis: the xenograft model studies have demonstrated through m^6^A sequencing that elevated METTL3 expression augments cellular migration, spheroid formation, and neoplastic proliferation by attenuating the translation of ABCD1 mRNA via an m^6^A-dependent pathway (Shi et al., 2021). ABCD1, a gene implicated in oncogenesis, when silenced, results in diminished migratory capacity and spheroid development in ccRCC cells. These findings highlight the crucial role of METTL3 in facilitating ccRCC progression through METTL3-ABCD1 interaction mechanism (METTL3↑ → m^6^A modification of ABCD1 mRNA → translational suppression → enhanced migration/spheroid formation). Additionally, the METTL3-HHLA2 Axis in ccRCC: the increased HHLA2 expression is associated with ccRCC advancement and adverse prognostic outcomes (Zhu et al., 2022). METTL3 regulates HHLA2 expression by promoting m^6^A modification of its mRNA, which in turn enhances the stability and abundance of HHLA2 mRNA (METTL3 ↑ → adds m^6^A modifications to HHLA2 mRNA→m^6^A stabilizes HHLA2 mRNA → increases its expression) (Zhu et al., 2022). Hence, METTL3 acts as an epigenetic driver of HHLA2, making it a potential prognostic biomarker. Moreover, the dual role of METTL3 in Kaposi’s sarcoma-associated herpesvirus (KSHV) infection: elevated METTL3 levels affect the oncogenic human DNA virus KSHV across various cell types (Hesser et al., 2018). In ccRCC cells, METTL3 knockdown significantly reduces virion production due to its involvement in the m^6^A modification of the primary viral lytic transactivator open reading frame 50 (ORF50), thereby impeding its post-transcriptional accumulation (METTL3 knockdown ↓ → reduces virion production by destabilizing m^6^A-modified ORF50 mRNA). Conversely, in KSHV-infected B cells, METTL3 depletion increases ORF50 protein levels, leading to enhanced virion production (METTL3 knockdown ↑ → increases ORF50 protein → boosts virion production) (Figures 1, 2; Table 1). Therefore, METTL3’s role in viral oncogenesis depends on cell type and m^6^A dynamics.

**TABLE 1:** Targets and functions of METTL3 in the pathogenesis of ccRCC.

METTL3		
Targets	Functions	References
*ABCD1 mRNA*	Attenuates *ABCD1* translation	Shi et al. (2021)
*HHLA2 mRNA*	Enhances *HHLA2* stability and abundance	Zhu et al. (2022)
*ORF50 mRNA*	Enhances *ORF50* post-transcriptional accumulation	Hesser et al. (2018)
*CAT, ERBB2, P2RY8, PLAUR, TIMP1 mRNA*	Enhances the translation and expression of *CAT, ERBB2, P2RY8, PLAUR, and TIMP1*	Ye et al. (2022)
*LAG3, TIGIT, CTLA4 mRNA*	Enhances the translation and expression of *LAG3, TIGIT, and CTLA4*	Zhang et al. (2022)
*GLUT1 mRNA*	Enhances *GLUT1* translation and expression	Zhan et al. (2023)
*DBT mRNA*	Promotes *DBT* degradation and decreases its expression	Miao et al. (2023)
*NCAPH mRNA*	Enhances *NCAPH* expression by augmenting its nuclear export and mRNA stability	Chen et al. (2023)
*LncRNA NEAT1*	Downregulates *LncRNA NEAT1* expression by diminishing its m^6^A methylation	Chen et al. (2021)

The m^6^A modification mediated by METTL3 plays a pivotal role in the pathogenesis of ccRCC, by regulating different RNAs.

Methyltransferase-like 3 (METTL3). N^6^-methyladenosine (m^6^A). Clear cell renal carcinoma (ccRCC). ATP-binding cassette subfamily D member 1 (ABCD1). Open reading frame 50 (ORF50). Glucose transporter 1 (GLUT1). Dihydrolipoamide branched chain transacylase E2 (DBT). On-SMC, condensin I complex subunit H (NCAPH).

#### 2.1.2 METTL3 functions by regulating immune and inflammation-related mRNAs

Tumor-infiltrating immune cells within the tumor microenvironment play a pivotal role in determining the progression of tumors (Mao et al., 2021; Virassamy et al., 2023; Li et al., 2023). Bioinformatics analysis has revealed that dysregulated fat mass and obesity-associated protein (FTO) and METTL3 may contribute to the development and progression of ccRCC by influencing immune responses (Zhao and Lu, 2021). A comprehensive data analysis conducted by Ye et al. (2022) demonstrated that METTL3-mediated m^6^A modification on CAT, ERBB2, P2RY8, PLAUR, and TIMP1 mRNA influences immune cell infiltration, subsequently promoting ccRCC progression. Moreover, inflammation-related regulators are implicated in the advancement and malignancy of ccRCC. Robust bioinformatics analysis confirmed an upregulation of m^6^A regulator METTL3 in the high-risk subgroup with a distinct inflammation-related gene signature. This upregulation functions through mediating m^6^A modification on LAG3, TIGIT, and CTLA4 mRNA to enhance their expression levels positively associated with risk score and indicative of high-risk status in individuals with ccRCC (Zhang et al., 2022). These findings collectively suggest that METTL3 serves as a predictive marker for ccRCC malignancy by regulating both immune infiltration and inflammation-related genes (Figures 1, 2; Table 1).

#### 2.1.3 METTL3 functions by regulating glycolysis-related mRNAs

Glucose transporter 1 (GLUT1) is a facilitative glucose transporter integral to tumor cell proliferation, survival, and chemoresistance (Rabbani et al., 2022). Zhan et al. (Zhan et al., 2023) elucidated that METTL3 augments GLUT1 expression, thereby accelerating ccRCC progression and conferring resistance to mammalian target of rapamycin (mTOR) inhibitors. Concurrently, dihydrolipoamide branched chain transacylase E2 (DBT) functions as a tumor suppressor by inhibiting tumor progression and rectifying lipid metabolism abnormalities in ccRCC. The investigation disclosed that METTL3-mediated m^6^A modification on DBT mRNA precipitates its downregulation, which activates Hippo signaling via ANXA2 interaction. This process diminishes the nuclear localization of yes-associated protein (YAP) and suppresses lipogenic gene transcription (Miao et al., 2023). Collectively, the aberrant downregulation of DBT induced by METTL3-mediated m^6^A modification facilitates ccRCC progression and lipid accumulation through augmented YAP nuclear localization. Therefore, targeting either YAP or DBT m^6^A modification is promising for therapeutic strategies against ccRCC. Furthermore, glycolysis plays a pivotal role in tumor immune evasion. Both *in vitro* and *in vivo* studies by Chen et al. (2023) have shown that non-SMC condensin I complex subunit H (NCAPH) upregulation in ccRCC promotes tumor cell proliferation, CD8^+^ T cell dysfunction, and programmed death-ligand 1(PD-L1) expression by inhibiting β-catenin degradation, ultimately enhancing aerobic glycolysis and immune tolerance in ccRCC. METTL3-mediated m^6^A modification on NCAPH augments its nuclear export and mRNA stability (Chen et al., 2023). Specifically, YTHDC1 facilitates NCAPH mRNA nuclear export while IGF2BP3 enhances its stability in an m^6^A-dependent manner. Collectively, NCAPH upregulation in patients with ccRCC correlates with poor therapeutic outcomes, indicating its potential oncogenic role. Additionally, NCAPH’s promotion of ccRCC growth and resistance to anti-PD-1 therapy underscores its potential as a prognostic predictor and immunomodulatory target, as well as an effective therapeutic strategy for this malignancy (Figures 1, 2; Table 1).

#### 2.1.4 METTL3 modulates tumorigenesis through lncRNA regulation

Beyond its role in mRNA modification, METTL3 also targets lncRNAs that are crucial for tumorigenesis and cancer progression, thus positioning them as innovative tumor markers (Tan et al., 2021; Xia et al., 2022; Vaid et al., 2024). One such lncRNA, nuclear enriched abundant transcript 1 (NEAT1), is markedly downregulated in ccRCC (Ghafouri-Fard and Taheri, 2019). Research indicates a relationship between the downregulation of NEAT1 and METTL3, resulting in diminished methylation of NEAT1 in ccRCC cells, which in turn facilitates tumor cell proliferation and migration (Chen et al., 2021). Significantly, reduced NEAT1 expression and m^6^A methylation levels are strong prognostic markers for patients with ccRCC. These observations imply that METTL3-mediated m^6^A methylation of NEAT1 may suppress NEAT1 expression, acting as a critical regulator of tumorigenesis and progression, and highlighting its potential as a therapeutic target for ccRCC treatment (Figures 1, 2; Table 1).

Collectively, METTL3 influences the initiation, progression, metastasis, poor prognosis, and malignancy of ccRCC through various mechanisms, including epithelial-mesenchymal transition (EMT), modulation of inflammatory responses, cell cycle regulation, alterations in glycolytic metabolism, immune evasion and infiltration strategies, and the modulation of cellular biological functions. These insights underscore the high-risk genetic attributes of METTL3 in ccRCC and bolster its potential utility as a diagnostic and prognostic biomarker for this malignancy.

### 2.2 METTL3 and drug resistance in ccRCC

The current standard treatment for ccRCC consists of a combination of surgical resection and targeted pharmacotherapies. Nonetheless, the development of drug resistance often complicates the therapeutic management of patients with ccRCC.

Tyrosine kinase inhibitors (TKIs), such as *Pazopanib*, are utilized as the first-line agents for managing metastatic ccRCC (Cheng et al., 2023). However, the effectiveness of *Pazopanib* is typically constrained to about 12 months, after which resistance inevitably develops. The resistance mechanism is hypothesized to involve METTL3-mediated m^6^A RNA modification. For example, during extended *Pazopanib* administration, the lnc RNA IGFL2-AS1 undergoes demethylation by the METTL3/METTL14 complex, leading to its stabilization due to reduced recognition by YTHDF2 (Cheng et al., 2023). This stabilized IGFL2-AS1 associates with the 5′-untranslated region of androgen receptor (AR) mRNA, inhibiting its degradation and promoting AR translation. Consequently, IGFL2-AS1 enhances *Pazopanib* resistance in ccRCC by interacting with AR mRNA. Thus, targeting the METTL3-IGFL2-AS1/AR axis may potentiate *Pazopanib’s* efficacy in curtailing ccRCC progression (Table 2).

**TABLE 2:** Targets and functions of METTL3 in ccRCC treatment.

METTL3			
Targets	Functions	Outcomes	References
*Lnc RNA IGFL2-AS1*	Demethylation and stabilization on Lnc RNA IGFL2-AS1 by reducing YTHDF2 recognition	Inhibits *AR mRNA* degradation and promotes *AR* translation to enhance *Pazopanib* resistance	Cheng et al. (2023)
*ALOX12, P53 mRNA*	Enhances the stability and translation of ALOX12 and P53 mRNAs	Reduces oxidative stress damage and ferroptosis within ccRCC cells	Shen et al. (2023)
*ZNF677 mRNA*	Stabilizes and translates *ZNF677 mRNA* via the recognition of IGF2BP2 and YTHDF1	Inhibits ccRCC progression by transcriptionally repressing *CDKN3 mRNA*	Li et al. (2022)
*HIF-2α*	Disrupts HIF-2α-driven metabolic adaptation	Inhibits advanced ccRCC	Zhan et al. (2023)
*PD-L1*	Enhances PD-L1 degradation via YTHDF2-mediated mRNA decay	Sensitizes ccRCC to anti-PD-1/PD-L1 therapies by influencing the tumor immune microenvironment	Zhan et al. (2023)

METTL3 contributes to the failure and treatment of ccRCC by mediating m^6^A modification on different RNAs.

Methyltransferase-like 3 (METTL3). N^6^-methyladenosine (m^6^A). Clear cell renal carcinoma (ccRCC). Androgen receptor (AR).YT521-B homology domain-containing family (YTHDF). Hypoxia-inducible factor 2α (HIF-2α). Programmed death-ligand 1(PD-L1).

Moreover, METTL3’s involvement in fostering *Pazopanib* resistance in ccRCC has been documented, yet its role in other forms of drug resistance remains unexamined. Therefore, comprehensive investigations are imperative to elucidate METTL3’s specific contributions to drug resistance mechanisms in ccRCC.

### 2.3 Targeting METTL3 for ccRCC therapy

Research has elucidated that METTL3 is anomalously overexpressed in ccRCC and is instrumental in driving tumor metastasis, metabolic reprogramming, and resistance to anticancer therapies. Consequently, METTL3 presents as a viable therapeutic target for ccRCC. Emerging strategies targeting METTL3 include small-molecule inhibitors, RNA-based therapies, and synergistic approaches with existing anticancer agents, all of which hold significant translational potential.

#### 2.3.1 Small-molecule inhibitors and phytochemicals

*Erianin*, a phytochemical extracted from dendrobium chrysotoxum, has shown efficacy in curtailing the proliferation, invasion, angiogenesis, and tumorigenesis of ccRCC. Shen et al. (2023) elucidated that *Erianin* downregulates METTL3 while concomitantly upregulating FTO, thereby enhancing the stability and translation of ALOX12 and P53 mRNAs. This modulation reduces oxidative stress damage and ferroptosis within ccRCC cells, offering a novel mechanism to overcome therapeutic resistance. Notably, *Erianin’s* ability to target both METTL3 and FTO highlights its potential as a multifaceted agent for ccRCC management (Table 2).

#### 2.3.2 Tumor suppressor pathways and m^6^A-Mediated regulation

The zinc finger protein ZNF677, a zinc finger protein family member, a tumor suppressor in ccRCC, is epigenetically regulated by METTL3. Li et al. (2022) revealed that METTL3 stabilizes ZNF677 mRNA via IGF2BP2- and YTHDF1-dependent m^6^A modifications, enhancing its translation. Elevated ZNF677 levels transcriptionally repress CDKN3 (cyclin-dependent kinase inhibitor 3), a driver of cell cycle progression, thereby inhibiting ccRCC proliferation and metastasis. This pathway underscores the therapeutic value of METTL3 modulation in restoring tumor suppressor activity (Table 2).

#### 2.3.3 Emerging clinical trials and combination therapies

The clinical relevance of METTL3 is further amplified by its role in patient stratification. High METTL3 expression correlates with advanced tumor stage, poor prognosis, and resistance to TKIs in ccRCC, positioning it as a predictive biomarker. Recent study is evaluating METTL3-targeting agents, including STM2457—a selective METTL3 inhibitor—in advanced ccRCC (Shaikh et al., 2025). Preclinical data suggest that METTL3 inhibition synergizes with mTOR inhibitors (e.g., everolimus) by disrupting hypoxia-inducible factor-2α (HIF-2α)-driven metabolic adaptation, a hallmark of ccRCC (Zhan et al., 2023) (Table 2).

#### 2.3.4 Immunomodulatory potential

METTL3 also influences the tumor immune microenvironment. Its suppression enhances PD-L1 degradation via YTHDF2-mediated mRNA decay, potentially sensitizing ccRCC to anti-PD-1/PD-L1 therapies (Zhan et al., 2023). This immunomodulatory effect, combined with metabolic reprogramming, positions METTL3 as a linchpin for combination regimens integrating immunotherapy and epitranscriptomic modulation (Table 2).

In summary, current therapeutic strategies targeting METTL3 emphasize precision medicine, leveraging its dual role as a biomarker and a druggable node. Future research should prioritize optimizing METTL3 inhibitors for clinical use, validating combinatorial approaches, and exploring resistance mechanisms. With the advent of m^6^A-focused therapies, METTL3 remains at the forefront of ccRCC translational research.

## 3 Discussion

METTL3 emerges as a central orchestrator of ccRCC pathogenesis, driving tumorigenesis, metastasis, and therapy resistance through its RNA m^6^A-modifying activity. This review posits that METTL3 disrupts renal cell fate and is instrumental in the onset and advancement of ccRCC. By regulating key processes such as metabolic reprogramming (e.g., lipid metabolism, glycolysis), immune evasion, and EMT, METTL3 stabilizes pro-tumorigenic transcripts (e.g., HIF-1α, vascular endothelial growth factor [VEGFA]) while destabilizing tumor suppressors (e.g., ZNF677), thereby enabling ccRCC proliferation and invasion. Clinically, METTL3 overexpression correlates with advanced tumor stage, poor prognosis, and resistance to TKIs, solidifying its dual utility as a diagnostic/prognostic biomarker and a therapeutic target. Therapeutic strategies targeting METTL3—including pharmacologic inhibition (e.g., *Erianin*, STM2457) and combination regimens with mTOR or immune checkpoint inhibitors—exploit its role in ccRCC’s molecular vulnerabilities. For instance, METTL3 suppression synergizes with anti-PD-1 therapies by enhancing PD-L1 degradation and T-cell infiltration. Therefore, addressing context-dependent METTL3 functions (e.g., tumor stage-specific roles) and resistance mechanisms will be critical to translating these insights into precision therapies.

## 4 Future perspectives

This review emphasizes the current research limitations regarding METTL3 modifications in ccRCC. For instance, the precise mechanism underlying METTL3’s involvement in ccRCC drug resistance remains incompletely elucidated. Additionally, while inhibition of METTL3 exhibits promising effects for treating ccRCC, there is a scarcity of specific inhibitors targeting METTL3 necessitating further development. Moreover, despite several studies demonstrating the potential therapeutic benefits of targeting regulatory factors associated with METTL3 and its signaling axes in ccRCC, substantial clinical data supporting this concept are still lacking. Therefore, while METTL3 has emerged as a pivotal player in ccRCC pathogenesis, critical knowledge gaps and challenges must be addressed to advance its therapeutic targeting. For example, recent studies reveal conflicting evidence about METTL3’s role across cancer types. In ccRCC, METTL3 is predominantly oncogenic, driving m^6^A-dependent stabilization of HIF-2α and VEGFA to promote angiogenesis and metastasis (Cheng et al., 2023; Chen et al., 2024a; Zhu et al., 2023; Shi et al., 2024; Li et al., 2025). However, in other malignancies (e.g., glioblastoma), METTL3 exhibits tumor-suppressive effects by stabilizing PTEN mRNA (Liu et al., 2023; Zha et al., 2020). This duality underscores the need to define tissue-specific METTL3 functions and regulatory networks in ccRCC, particularly across disease stages (early vs metastatic) and molecular subtypes (e.g., risk groups). Additionally, the ccRCC microenvironment (e.g., hypoxia, lipid-rich niches) uniquely shapes METTL3 activity. For instance, hypoxia induces METTL3 to stabilize HK2 mRNA, amplifying glycolysis (Jung et al., 2024; Chen et al., 2024b; Xu et al., 2021; Li et al., 2017; Zhu et al., 2025). Comparative studies profiling METTL3 targets in ccRCC *versus* normal kidney tissue could uncover tumor-specific vulnerabilities. Similarly, exploring METTL3’s role in ccRCC-associated fibroblasts or immune cells may reveal novel stromal targets.

To address these gaps, it is essential to further investigate the implications of targeting METTL3 and its related pathways. The therapeutic potential of METTL3 is highlighted by its involvement in RNA methylation, which influences a variety of cellular processes, including gene expression, cell proliferation, and apoptosis. Gaining a deeper understanding of how METTL3 modifies target RNAs could shed light on its role in ccRCC drug resistance, particularly concerning conventional therapies that frequently lead to treatment failures. The mechanisms through which METTL3 contributes to drug resistance are likely complex; for example, METTL3 may regulate the expression of genes that are pivotal in drug metabolism and cellular stress responses, consequently affecting the sensitivity of ccRCC cells to chemotherapeutic agents. Recent research has shown that m^6^A modifications facilitated by METTL3 can stabilize certain oncogenes while promoting the degradation of tumor suppressor mRNAs. This dual function may foster a cellular environment that is favorable for tumor survival and growth, especially under therapeutic pressure. Therefore, identifying the specific mRNA targets of METTL3 in ccRCC could reveal pathways that, when inhibited, may reverse resistance and improve the effectiveness of current treatments.

Moreover, the advancement of targeted METTL3 inhibitors is vital for translating these research insights into clinical practice. Existing pharmacological strategies aimed at inhibiting RNA methylation lack selectivity and may inadvertently affect other methyltransferases, resulting in off-target effects. Thus, a strategic drug design approach that focuses on the distinctive structural characteristics of METTL3 is essential. Innovative high-throughput screening methods could facilitate the discovery of selective inhibitors, reducing potential side effects and enhancing therapeutic efficacy. In terms of clinical significance, establishing biomarkers linked to METTL3 activity could greatly improve patient stratification in ccRCC. Identifying patients with elevated METTL3 expression or specific METTL3-dependent m^6^A modifications could guide treatment choices and anticipate therapeutic responses. Furthermore, longitudinal investigations into the dynamics of METTL3 modifications during treatment could yield valuable insights into tumor evolution and adaptation, paving the way for more tailored therapeutic approaches.

The interplay between METTL3 modifications and other signaling pathways in ccRCC also merits further investigation. For instance, exploring the interactions between m^6^A methylation and pathways such as mTOR or HIF signaling could uncover synergistic relationships that promote tumor growth and metastasis. Understanding these connections may lead to combination therapies that target multiple pathways, thereby addressing the limitations of monotherapy and the intricate nature of ccRCC. In summary, bridging the knowledge gaps surrounding METTL3 in ccRCC is crucial for advancing treatment strategies. Future research should not only aim to clarify the mechanistic roles of METTL3 but also prioritize the development of selective inhibitors and the identification of biomarkers. By integrating fundamental research with clinical applications, we can more effectively leverage the potential of targeting METTL3 to enhance outcomes for patients with ccRCC.

## 5 Conclusion

In conclusion, this review clarifies the relationship between METTL3 and ccRCC, offering a thorough understanding of the underlying mechanisms. We further propose that METTL3 shows promise as a diagnostic and prognostic biomarker for ccRCC, providing valuable insights for both researchers and clinicians.
